# Position paper on symbiotic intelligence in healthcare: Can AI help us better understand suicidal behavior and prevent suicide?

**DOI:** 10.3389/fmed.2026.1848732

**Published:** 2026-06-22

**Authors:** Rune Johan Krumsvik, Kjetil Laurits Høydal, Øyvind Arne Høydal

**Affiliations:** 1 Faculty of Psychology, University of Bergen, Bergen, Norway; 2 Faculty of Arts and Physical Education, Volda University College, Volda, Norway; 3 Faculty of Humanities and Education, Volda University College, Volda, Norway

**Keywords:** alcohol use, artificial intelligence, conceptual model, ecological momentary assessment, social media exposure, suicide prevention, symbiotic intelligence, wearable sensors

## Abstract

Suicide is the second leading cause of death among young people aged 10–24 worldwide, yet identifying individuals at risk remains a major challenge. In Norway, suicide rates reached their highest level in 25 years in 2024, underscoring persistent knowledge gaps in national prevention efforts and the need for innovative, interdisciplinary, and supplementary approaches. There are no simple solutions to mental ill health or suicide, and no single factor can adequately explain such complex phenomena; however, it remains crucial to examine how interacting risk factors may increase vulnerability and whether artificial intelligence can help identify emerging risk windows earlier, thereby complementing conventional clinical approaches in this field. The current evidence base suggests a need for increased vigilance in several areas, particularly regarding Gen Z's digital lifestyle, exposure to shock-like societal events, and patterns of alcohol consumption, as these factors may interact in ways that elevate suicide risk. The rapid growth of social media use over the past decade have given both a *Werther-* and *Papageno-*effects, raising questions about both the positive effects of social media use and also whether certain patterns of social media engagement may contribute to suicide risk among vulnerable groups. Emerging evidence indicates that shock events in society and extreme media exposure may affect vulnerable individuals indirectly and without conscious awareness, thereby increasing short-term suicide risk. Research from Norway and other countries shows that traumatic events may trigger acute spikes in suicides, influence perinatal outcomes, and affect population-level health indicators such as sex ratios and infant mortality. These “triple-hit” patterns suggest that indirect exposure through media may be more consequential than previously assumed. Additionally, alcohol use may play a significant role in short-term risk escalation by increasing impulsivity, reducing cognitive control, and intensifying emotional distress. International studies show that alcohol use is significantly associated with increased suicidality, and in Norway approximately four in ten individuals who die by suicide have alcohol in their bloodstream at the time of death. Early-warning systems could thus benefit from integrating alcohol-related indicators. This position paper argues that three opportunities are particularly salient. First, the majority of primary studies within this area rely on conventional research designs and analytical approaches, with limited use of artificial intelligence-supported methods that could potentially enhance measurement precision, validity, and reliability in the analysis of complex digital behaviors. For example, AI-based linguistic analysis of social media content may help detect short-term “risk windows” associated with psychological distress, depression, and suicidality. Second, improved access to anonymized, high-quality platform data from technology companies could strengthen population-level monitoring and research. Third, actigraphy and AI integrated with wearable sensors and brief daily ecological momentary assessments (EMA) may capture subtle fluctuations in sleep, stress, heart rate, and activity—patterns that often precede clinical deterioration but may go unnoticed by patients, families, and clinicians. While international studies suggest that AI can enhance short-term risk detection, such systems must neither replace human contact nor override core principles of privacy, consent, and autonomy. Rather, AI should function as a complementary, real-time alert layer (symbiotic intelligence) capable of informing timely and tailored interventions within existing health services. Given current knowledge gaps and the rising impact of shock-related stressors, health authorities should consider piloting AI-supported early-warning systems that are tightly embedded in clinical pathways, e.g., through a new conceptual model presented in this paper. AI alone will not save lives, but small, ethically grounded steps may help identify individuals in rapidly escalating distress before it is too late.

## Introduction

Suicide is a highly complex phenomenon, and existing evidence indicates a continued need for innovative prevention and intervention approaches. Suicide is the second leading cause of death among young people aged 10–24 worldwide (1). However, identifying individuals at risk remains a major challenge.

In recent years, despite national action plans and prevention campaigns, suicide rates in Norway have developed in an unfavorable direction. The number of suicides in 2024 reached 739—the highest recorded since 1999—and nearly two out of three were men (2). The Norwegian Institute of Public Health (3) has highlighted substantial knowledge gaps in current prevention strategies and emphasized the need for both small- and large-scale multidisciplinary and cross-sectoral interventions.

One partly unexplored aspect of suicide prevention concerns the role of shock events and extreme media exposure. It may be important to examine how such exposure affects vulnerable groups and whether small AI-supported monitoring or early-warning measures could contribute to prevention efforts. Although this may appear to be a relatively marginal field within suicide prevention, both Norwegian and international research point to concerning tendencies that warrant closer attention (4).

At the same time, the rapid growth of social media use has coincided with increasing levels of suicidal thoughts and behaviors (STBs). This raises questions about whether certain forms of digital engagement may contribute to suicide risk or reflect emerging psychological distress among vulnerable individuals (5). These developments highlight the potential value of interdisciplinary approaches capable of detecting early signals of escalating distress, including the use of artificial intelligence to analyse digital behavior, language patterns, and real-time behavioral data.

Alcohol use is another factor that deserves greater attention in suicide prevention. Norwegian data indicate that approximately four in ten individuals who die by suicide have alcohol in their bloodstream at the time of death (6). Alcohol intoxication may increase impulsivity, reduce cognitive control, and intensify emotional distress. This may narrow the time window between suicidal ideation and suicidal action. Despite this, alcohol use is often under-addressed in discussions about the early detection of suicide risk.

Integrating alcohol-related indicators into early-warning systems may therefore represent an additional avenue for identifying periods of elevated risk. Such indicators could include self-reported alcohol consumption, behavioral changes, or physiological signals detected through wearable devices. International systematic reviews and meta-analyses consistently indicate that alcohol use is associated with increased suicide risk. For example, one meta-analysis reported a 65% higher suicide risk associated with alcohol use (7). Another meta-analysis found that alcohol use disorder (AUD) was significantly associated with increased suicidality (8). Acute alcohol consumption appears to be particularly important, with one meta-analysis reporting nearly sevenfold higher odds of suicide attempts following alcohol use (9). At the population level, higher per capita alcohol consumption has also been associated with increased suicide mortality rates (10).

In Norway approximately 41% of students report risky or harmful alcohol use and additional data indicate that around 8–11% have an alcohol use disorder. Approximately 1 in 5 students have experienced suicidal thoughts among student in higher education (11). While this study is descriptive, the patterns observed suggest that alcohol consumption may interact with underlying psychological vulnerabilities, potentially contributing to short-term escalation of suicide risk.

The aim of this position paper is to explore whether AI-supported research designs, artificial intelligence, symbiotic intelligence and digital behavioral signals—such as language patterns, social media activity, wearable sensor data, and ecological momentary assessments (EMA)—can help detect emerging suicide risk earlier than traditional clinical assessments. In particular, the paper considers how interactions between alcohol use, media exposure, and digital behavior may create short-term “risk windows” that could potentially be identified through AI-supported early-warning systems (visualized through a concept model).

## Methods

This position paper is based on a conceptual and interdisciplinary synthesis of peer-reviewed literature within suicidology, public health, digital health, artificial intelligence, social media research, alcohol-related suicide risk, ecological momentary assessment, wearable sensing, and suicide prevention. The paper does not aim to provide a systematic review or meta-analysis. However, to reduce the risk of anecdotal selection, priority was given to recent systematic reviews, meta-analyses, large population-based studies, and theoretically relevant empirical studies. Literature was identified through targeted searches in PubMed/MEDLINE, PsycINFO, Scopus, Web of Science, and Google Scholar, supplemented by backward citation searching of relevant reviews. Search terms included combinations of: suicide, suicidal ideation, suicide prevention, artificial intelligence, machine learning, passive sensing, wearables, actigraphy, ecological momentary assessment, social media, media exposure, Werther effect, Papageno effect, alcohol use, digital health, and early warning systems. The synthesis was analytical and hypothesis-generating, with the aim of developing a conceptual model of AI-supported symbiotic intelligence in suicide prevention. In this paper, symbiotic intelligence refers to collaborative interaction between human clinical judgement and AI-supported analytical systems, where AI functions as a complementary decision-support and situational-awareness tool rather than an autonomous replacement for human care.

In addition, to address this issue more systematically in future work, we have developed a PRISMA-ScR-informed scoping review protocol that will map the evidence on AI-supported, wearable, EMA-based, social media-related, and alcohol-related approaches to identifying short-term suicide risk windows within a symbiotic intelligence framework (see Attachment 1).

## Can shock events and social media exposure affect vulnerable groups?

In several op-eds (12–14), scholarly articles (15–17), and a book (18), we have highlighted the need to remain vigilant regarding how the rapid expansion of technology and media exposure among Gen Z over the past 15 years may influence both educational outcomes and mental health. Ordinary technology and media use is, of course, an integral part of contemporary digital society and is generally unproblematic for most young people. However, when the scope, intensity, and content of exposure become extreme or shocking, caution is warranted. Such exposure may affect vulnerable groups in particular, including individuals experiencing suicidal ideation, pregnant women, and others. The international research literature suggests that traumatic events and extreme media exposure may act as triggering factors in individuals who already face an elevated risk of suicide, even if such exposures are not the primary underlying cause (4).

While editorially regulated media generally maintain a high level of caution when reporting on suicide-related issues, social media environments are far less controlled. Within these digital spaces, Gen Z may be exposed to a wide range of potentially disturbing or harmful content. Media use among young people is extremely high. Norwegian students, for example, spend an average of approximately 7 h per day on screen-based activities. The majority report between 3 and 9 h of daily screen time (71%), while as many as 26% report 10 h or more. At the same time, mental health problems are widespread among Norwegian students. Approximately 42% report mental health difficulties, and 35% experience symptoms that may be classified as severe (11, 19). At the same time, causal relationships between social media use and mental health problems have not been firmly established in these studies, which is also a consistent limitation across the broader international evidence base.

Internationally, a systematic review of 50 studies examining the relationship between screen time and adolescent mental health found that most studies reported associations between higher levels of screen exposure and poorer mental well-being. Smartphones were the most commonly used devices, and increased weekday screen use was associated with reduced well-being. Social media use in particular was linked to poorer mental health outcomes, and among girls it was associated with a higher risk of depression (20). Notably, however, such associations do not imply causation.

Fassi et al. (65) finds that this area has high complexity. The study shows that adolescents with mental health conditions spend more time on social media than their peers, but usage patterns differ by condition type. Those with internalizing conditions (e.g., anxiety and depression) are more affected by social comparison and feedback, and report lower satisfaction with friendships and self-disclosure. Adolescents with externalizing conditions mainly differ in increased time spent. The findings highlight the need for a more nuanced understanding of the relationship between social media and mental health, and for tailored approaches in policy and clinical practice.

Overall, the area is complex and even if some findings suggest that excessive screen time may be associated with mental health problems among some vulnerable groups among adolescents, further research is needed to clarify the role of specific types of screen use and digital content (20) (Fassi et al., 2025). One reason for this concern is the possibility that shock-like impressions may sometimes operate “under the radar,” exerting psychological stress effects without individuals being fully aware of them. In recent articles (21–23), we therefore discuss whether it may be useful to examine more closely what artificial intelligence could potentially contribute to the monitoring and understanding of various aspects of mental health in the population.

An extensive digital lifestyle among Gen Z can be related to other risk factors for some vulnerable groups. International research suggests that university students represent a Gen Z-group with relatively high levels of alcohol consumption, often shaped by social norms and drinking cultures within higher education environments (24–26). Increased vigilance regarding the relationship between alcohol use and other interacting risk factors among vulnerable Gen Z student populations is therefore becoming increasingly important for understanding this phenomenon.

Taken together, these observations raise the possibility that large-scale societal shock events, intense (social) media exposure and alcohol use may have broader and sometimes unintended consequences for population health, particularly among already vulnerable individuals.

One illustrative example of such potential population-level effects has been discussed in the Norwegian context following the terrorist attacks of 22 July 2011 described below.

## Norway experienced a “triple hit”

Shock events and intense media exposure may affect not only those who experience such events directly, but also individuals who are indirectly exposed through media coverage, social networks, and broader societal reactions. A relevant and sobering example has been discussed in a knowledge overview by Masukume et al. (4), which suggests that Norway may have experienced a possible “triple hit” following the terrorist attacks of 22 July 2011: the killing of 77 people, a temporary increase in suicides in the subsequent period, and changes in perinatal outcomes, including increased infant mortality.

A Norwegian study reported a 105% increase in suicides during the first week following the attack and a 45% increase during the subsequent 4 weeks (27). While such patterns do not establish direct causal relationships, they raise important questions about how large-scale traumatic events and intense media exposure may interact with existing vulnerabilities within populations.

These observations illustrate how indirect exposure to large-scale societal trauma may potentially influence population-level health outcomes beyond the immediate victims of the event.

## Infant mortality and sex ratios are affected

Other vulnerable groups may also be affected by large-scale shock events. A number of studies suggest that such events may influence perinatal outcomes, including infant mortality. For example, Masukume et al. (4) reported that the number of male fetuses lost during pregnancy following the 22 July 2011 terrorist attacks in Norway was substantially higher than expected, suggesting that maternal stress associated with the attacks may have contributed to miscarriages or stillbirths. An analysis of infant mortality among boys in the weeks following the attacks also indicated a marked increase in August 2011 (28).

These findings have been discussed in relation to the *Trivers–Willard hypothesis* (29), which proposes that severe maternal stress during pregnancy may influence offspring sex ratios, often resulting in relatively fewer male births. Although the underlying mechanisms remain debated, several studies have reported similar patterns following large-scale stress events.

Multiple reviews suggest that major shock events—including earthquakes, terrorist attacks, natural disasters (30), and pandemics (31)—may influence population-level health indicators such as birth outcomes and sex ratios (32). These findings highlight how large-scale societal stressors may affect not only those directly exposed but also individuals indirectly affected through broader social and psychological mechanisms.

## The Werther- and Papageno-effects

A related body of research suggests that media portrayals of suicide and exposure to suicide-related content may influence suicide rates in the short term, particularly following highly publicized cases or detailed reporting of suicide methods. Systematic reviews and meta-analyses have documented what are commonly referred to as the *Werther effect* and the *Papageno effect*, whereby certain forms of suicide-related media exposure may increase suicidal behavior, while narratives emphasizing coping strategies and recovery may have protective effects (33, 62).

For example, a meta-analysis of 31 studies found that suicide rates increased by approximately 13% following media reports of a celebrity suicide. When the specific suicide method was reported, deaths by the same method increased by about 30% during the following weeks (33).

A systematic review of 46 studies reported mixed associations between internet use and self-harm or suicidal behavior. While some studies reported protective effects and others harmful effects, negative outcomes were most often associated with internet addiction, high levels of online activity, and exposure to self-harm or suicide-related content (34).

Similarly, a systematic review and meta-analysis examining social media use and self-injurious thoughts and behaviors (SITB) found that individuals' engagement with suicide- and self-injury-related content on social media—both producing and consuming such content—may reinforce offline suicide and self-injury risk (35).

A systematic review and meta-analysis by Cabezas-Klinger et al. (36) also suggested potential implications for prevention. In one dataset, approximately 40% of adolescents who died by suicide had developed online identities centered around suicidal thoughts, indicating that digital behavior may provide early signals of escalating risk.

More broadly, a systematic review of 46 studies found that several aspects of social media use were associated with suicidal thoughts and behaviors (STBs), although most associations were relatively small. Higher overall use of social media and smartphones showed weak positive associations with STBs, while smartphone addiction demonstrated stronger associations. Suicide-related social media content and behaviors such as sexting were also positively associated with STBs, whereas findings regarding social media addiction were mixed (5).

A systematic search of studies published up to January 2019 across five major databases identified nine independent studies examining the relationship between social media or internet use and suicide attempts among individuals younger than 19 years (*n* = 346,416). Seven studies reported an independent association between heavy social media or internet use and increased suicide attempts, with adjusted odds ratios ranging from 1.03 to 5.10. However, these associations were substantially attenuated when factors such as cyberbullying victimization and sleep disturbances were considered. Two studies suggested that moderate levels of online engagement, compared with no use, might be associated with fewer suicide attempts. Notably, no studies examined the relationship between social media or internet use and completed suicide (1).

A systematic review and meta-analysis including 126 studies and more than 1.4 million adolescents found that social media use was associated with a range of health risk behaviors. Frequent social media use was linked to higher odds of alcohol consumption, drug and tobacco use, gambling, sexual risk behaviors, antisocial behavior, and multiple risk behaviors. Exposure to social media content depicting such behaviors was also associated with increased likelihood of engaging in them. Overall, the findings suggest that social media use may be associated with adverse health risk behaviors among adolescents, although the strength of the evidence varies and causality remains uncertain (37).

Similarly, a meta-analysis of 27 studies including more than 67,000 adolescents found small-to-moderate positive associations between social media use and engagement in risky behaviors. Social media use was associated with higher levels of substance use and risky sexual behavior, with stronger associations observed among younger adolescents and in studies examining a broader range of contemporary social media platforms (38).

Another systematic review and meta-analysis of 30 studies involving nearly 20,000 participants found that both exposure to alcohol-related content and posting such content on social networking sites were associated with higher alcohol consumption among young people. Cross-sectional analyses showed that greater exposure to- and self-posting of alcohol-related content in social media were linked to increased drinking levels, while prospective studies indicated that exposure to alcohol-related content predicted higher alcohol consumption over time (39).

Taken together, these studies suggest that the *Werther effect*—where exposure to suicide-related media coverage is associated with increased suicidal behavior—may also occur in digital environments. At the same time, the *Papageno effect* highlights how narratives focusing on coping strategies, recovery, and help-seeking may have protective influences. These contrasting mechanisms indicate that media environments may also represent an important arena for preventive communication and intervention.

Overall, a growing body of systematic reviews suggests that social media use may be associated with suicidal ideation, self-harm, and other self-injurious thoughts and behaviors. These associations appear particularly strong for exposure to self-harm content and cyberbullying, while overall time spent on social media shows weaker and more inconsistent relationships with suicide risk. Importantly, research also indicates that social media environments may produce both harmful contagion effects (the Werther effect) and protective influences (the Papageno effect), depending on the nature of the content and user interactions (34–36).

Taken together, these findings suggest that self-harm and suicide risk may be shaped by a complex interaction of societal shocks, media exposure, digital environments, and behavioral risk factors such as alcohol use. In highly connected societies, individuals may be exposed to emotionally intense information streams that can amplify stress reactions and reinforce vulnerability among already at-risk groups. These dynamics may create short-term “risk windows” during which suicidal behavior becomes more likely. However, such fluctuations in psychological distress are often difficult to detect using traditional clinical approaches. Emerging digital methods—including artificial intelligence, passive sensing technologies, and ecological momentary assessments—may therefore offer new opportunities to identify subtle behavioral and physiological changes associated with escalating risk in near real time.

A recurring limitation in the literature is that many primary studies analyse social media exposure at an aggregated level, “zooming out” to platforms in general rather than “zooming in” on specific environments such as Instagram, TikTok, or other platform-specific ecosystems. Another challenge is that the majority of primary studies rely on conventional research designs and analytical approaches, with limited use of artificial intelligence—supported methods that could potentially enhance measurement precision, validity, and reliability in the analysis of complex digital behaviors. Therefore, it is important to design studies examining whether AI-supported linguistic analysis of social media content may help identify markers associated with psychological distress and suicidality. For example, Wang et al. (40) demonstrated that deep-learning analysis of suicide-related social media posts was associated with real-world suicide patterns over a 10-year period in Japan, while Coppersmith et al. (41) and Eichstaedt et al. (42) showed that natural language processing approaches may detect signals associated with suicide risk and suicide attempts.

**Figure 1:**
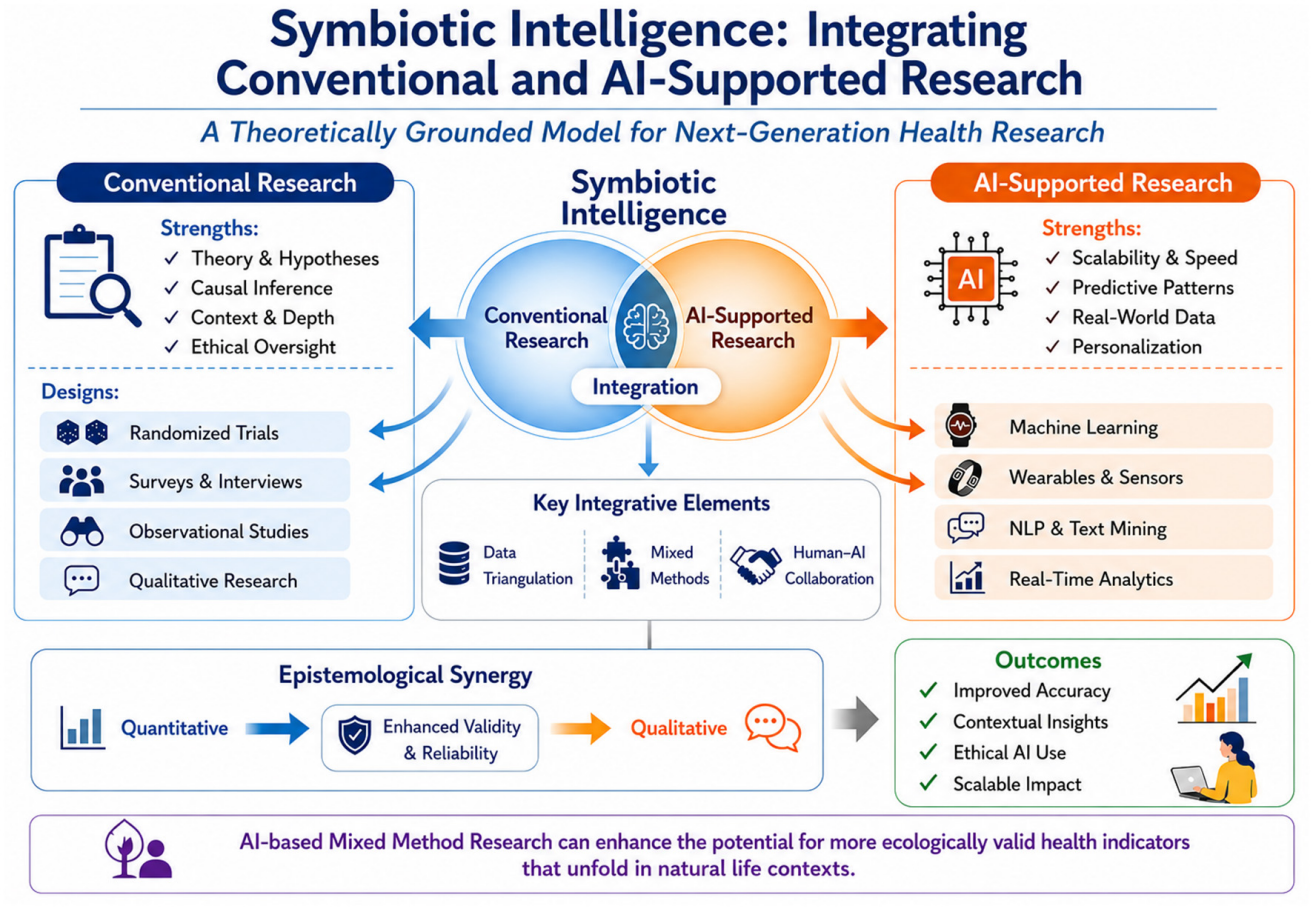
Illustrates a theoretically grounded model of symbiotic intelligence in research, where conventional and AI-supported research designs are integrated into a unified framework. Where conventional research contributes theory-driven inquiry, causal inference, contextual depth, and ethical oversight (e.g., through randomized trials, surveys, observational, and qualitative studies), AI-supported approaches provide scalability, speed, and complex pattern detection (e.g., through machine learning, real-time analytics, and data from wearables). Integration of these approaches, through data triangulation, mixed methods, and human–AI collaboration fosters epistemological synergy that bridges quantitative and qualitative traditions.

It seems increasingly necessary to develop more comprehensive and theoretically grounded research models in this thematic area, capable of integrating conventional methodologies with AI-supported approaches to ensure validity, robustness, and contextual sensitivity. Figure 1 illustrates a theoretically grounded model of symbiotic intelligence in research, where conventional and AI-supported research designs are integrated into a unified framework.

On the one hand, conventional research contributes theory-driven inquiry, causal inference, contextual depth, and ethical oversight through established designs such as randomized trials, surveys, observational, and qualitative studies. On the other hand, AI-supported approaches provide scalability, speed, and the capacity to identify complex patterns through machine learning, real-time analytics, and data from wearables and other digital sources. At the core of the model lies the integration of these approaches, operationalized through key mechanisms such as data triangulation, mixed methods, and human–AI collaboration. This integration fosters an epistemological synergy that bridges quantitative and qualitative traditions, enhancing validity, reliability, and contextual understanding. Overall, the model illustrates a possible shift from a dichotomous view of research methodologies toward a collaborative paradigm, where symbiotic intelligence enables more robust, scalable, and ethically informed research outcomes.

## Can artificial intelligence contribute?

Preventive measures aimed at shielding individuals from extreme societal and media events have often been sporadic and largely based on conventional interventions and research designs. Although some of these issues have occasionally been raised in public debate—for example in the Norwegian national newspaper *Dagbladet* in 2012 (60), where attention was drawn to the potential psychological effects of large-scale media exposure—there remains a clear need to strengthen the empirical knowledge base and explore innovative approaches to suicide prevention.

One question that therefore arises is whether emerging digital technologies, particularly artificial intelligence (AI), may contribute to identifying early signals of escalating distress. Rather than replacing established prevention strategies, small and carefully implemented AI-based approaches could potentially serve as complementary tools for detecting short-term changes in risk.

Three opportunities appear particularly relevant.

First, research suggests that AI-supported research designs with e.g., AI-based linguistic analysis of social media content may help detect patterns of psychological distress, depression, and short-term “risk windows” associated with suicidal ideation, etc. (40–42).

Second, improved access to anonymized, high-quality platform data from technology companies could strengthen population-level monitoring and research on suicide risk in digital environments.

Third, emerging studies within symbiotic intelligence indicate that AI combined with wearable technologies may provide new opportunities for monitoring short-term behavioral and physiological changes. Signals such as sleep patterns, stress levels, physical activity, and heart rate may fluctuate before psychological crises become clinically visible. Such changes often remain “under the radar” for both individuals and their close networks but may potentially be detected through continuous digital monitoring.

Against this background, a growing body of research has begun to explore how artificial intelligence, symbiotic intelligence, machine learning, wearable sensors, and ecological momentary assessments can contribute to the early detection of suicide risk. The following section therefore reviews recent empirical studies and systematic reviews examining the potential—and limitations—of AI-based approaches in suicide prevention.

## Can symbiotic intelligence and AI co-intervene when risk increases?

In recent years, a rapidly growing body of research has examined whether artificial intelligence and digital sensing technologies can help identify early warning signs of suicidal thoughts and behaviors. Recent reviews and empirical studies suggest that artificial intelligence, symbiotic intelligence, machine learning, wearable technologies, and actigraphy may offer new opportunities for detecting early warning signs of psychological distress. Continuous monitoring of physiological and behavioral signals—such as heart rate, sleep patterns, stress indicators, pulse, and physical activity—combined with brief daily ecological momentary assessments (EMA) may provide valuable real-time insights into fluctuations in mental health (43, 44). Such approaches raise the possibility of identifying subtle changes in behavioral and physiological patterns before individuals themselves recognize or articulate escalating distress, thereby potentially enabling earlier intervention during “critical time windows” when suicide risk may increase.

A scoping review of 42 studies examined the use of passive sensing from smartphones and wearable devices combined with machine learning to monitor mental health conditions such as depression and anxiety. The findings suggest that physiological and behavioral data—particularly heart rate, physical activity, sleep patterns, and indicators of social interaction—can be used to detect mental health changes with relatively high predictive accuracy. However, most studies were based on small samples and short monitoring periods, with limited external validation and insufficient attention to ethical issues such as data privacy. Overall, passive sensing and machine learning show promising potential for continuous, objective monitoring of mental health, but larger longitudinal studies and stronger methodological standards are needed before widespread clinical implementation (45).

A systematic review and meta-analysis of 42 studies including more than 1.4 million adolescents evaluated the performance of machine learning models for predicting suicide-related behaviors. The results showed that machine learning approaches demonstrated moderate to high predictive accuracy, particularly for suicide attempts. Ensemble methods such as random forest and gradient boosting performed especially well. However, most studies relied on internal validation and limited datasets, which may reduce the generalisability of the findings. Overall, machine learning shows promising potential for improving early identification of suicide risk in adolescents, although larger and externally validated studies are needed before widespread clinical application (46).

A systematic review of 41 studies evaluated the use of machine learning algorithms to predict suicide risk. The findings showed that several algorithms demonstrated moderate to high predictive performance, with random forest and gradient boosting models achieving the highest accuracy. The studies also identified common suicide risk factors, including age, gender, depression, anxiety, substance abuse, alcohol consumption, and socioeconomic variables. Although machine learning shows potential for improving suicide risk prediction, further research and clearer ethical guidelines are needed before these approaches can be widely implemented in clinical settings (47).

A systematic review by Büscher et al. (48) examined whether passive sensing data from smartphones and wearable devices can help predict suicidal thoughts and behaviors. The review found that while such technologies are feasible and show promise for monitoring behavioral and physiological signals associated with suicide risk, current evidence for their predictive value remains limited due to methodological weaknesses and small sample sizes.

A systematic review and meta-analysis of 53 studies evaluated the accuracy of machine learning algorithms in predicting suicide and hospital-treated self-harm. The models showed moderate predictive performance, with AUC values ranging from 0.69 to 0.93 and high specificity but variable sensitivity. However, due to the low base rate of suicide, the positive predictive value of these models remained low in most populations. The findings suggest that current machine learning algorithms are not sufficiently accurate for population-level screening or for prioritizing individuals for interventions. Instead, clinical care should continue to focus on comprehensive assessment and evidence-based aftercare for individuals presenting with self-harm (49).

Taken together, recent systematic reviews suggest that AI, digital technologies and machine learning may improve the early detection of suicide risk. Studies using passive sensing data from smartphones and wearable devices indicate that behavioral signals—such as sleep patterns, mobility, and activity levels—may help identify emerging suicidal thoughts and behaviors. At the same time, meta-analyses of machine learning models show promising predictive performance, although the overall evidence base remains limited by methodological heterogeneity, small samples, and insufficient external validation. Another caveat is that suicide is a low base-rate phenomenon; while many models show moderate discrimination, low prevalence limits positive predictive value in real-world settings. These findings highlight both the potential and the current limitations of AI-based approaches in suicide prevention.

## Ecological momentary assessment (EMA), actigraphy and AI

Combining ecological momentary assessment (EMA) with artificial intelligence is another promising area of research. A diagnostic study examined whether daily mood fluctuations and stressful events could predict suicidal ideation among sexual and gender minority young adults. Using EMA data collected twice daily over 25 days and analyzed with machine learning, the study found that dynamic daily experiences predicted suicidal ideation more accurately than baseline risk factors alone. The EMA-based models achieved good predictive performance ([[I]]AUC[[/I]] ≈ 0.80) for short-term suicidal ideation and remained moderately predictive at 3- and 8-month follow-ups. The findings highlight the importance of real-time contextual factors in suicide risk and suggest that combining EMA with machine learning may support earlier identification of individuals at risk (50).

Another study combining ecological momentary assessments, wearable data, and machine learning developed personalized models to predict depressed mood over a 1-month period. The findings revealed that optimal predictive performance was highly individualized across machine learning models, underscoring the significant heterogeneity inherent in depression dynamics. Key predictors of mood included factors such as anxiety, stress, sleep patterns, physical activity, diet, and cognitive performance. These findings suggest that integrating real-time behavioral and physiological data with machine learning may enable personalized monitoring and treatment strategies for depression (51).

A study of 78 suicidal adolescent inpatients used daily mobile surveys based on EMA over 4 weeks after hospital discharge to predict next-day suicidal ideation. Machine learning models analyzing dynamic daily risk factors—such as hopelessness, perceived burdensomeness, and self-efficacy—showed good predictive performance, with the best model achieving an AUC of 0.86. The findings suggest that combinations of real-time psychological indicators can help identify short-term increases in suicide risk and may support the development of digital tools for timely intervention (52).

An EMA study of 84 individuals with borderline personality disorder compared recurrent neural networks (RNNs) with traditional mixed-effects models for predicting daily suicidal ideation. Using repeated daily assessments of mood, stressful events, coping strategies, and affect, the results showed that RNNs achieved better predictive accuracy than conventional statistical models, particularly at higher levels of suicidal ideation. These findings suggest that deep learning methods may improve the analysis and prediction of suicidal thoughts using complex, time-varying EMA data (53).

A systematic review examined studies applying artificial intelligence to ecological momentary assessment data to predict suicidal ideation and behaviors. Twelve studies including nearly 4,400 participants were identified. The results showed that AI models demonstrated moderate predictive performance for near-term suicidal ideation, with AUC values ranging from 0.74 to 0.86. Although the findings highlight the promise of combining AI and EMA for real-time suicide risk detection, the evidence base remains limited and affected by methodological variability and inconsistent reporting practices (54).

More specifically, actigraphy-based research has examined how sleep patterns may be linked to suicidal ideation. An observational study using actigraphy found that more stable daily sleep–wake rhythms were associated with lower levels of suicidal ideation among older adults with depression, independent of depression severity. In contrast, sleep duration and fragmentation were less strongly associated with suicide risk. These findings suggest that disruptions in circadian rhythms may play an important role in suicidal ideation and could represent a potential target for preventive interventions (55).

Similarly, a longitudinal study combining ecological momentary assessment and actigraphy examined how sleep disturbances influence next-day suicidal ideation. Poor sleep quality, shorter sleep duration, and greater sleep fragmentation were associated with increased suicidal ideation the following day. Hopelessness partly explained the relationship between perceived insufficient sleep and suicidal ideation, suggesting that sleep disturbances may function as an early warning signal for elevated suicide risk (56).

An EMA study of high-risk adolescents examined the relationship between sleep patterns and next-day suicidal ideation using both actigraphy and sleep diaries. The results showed that longer sleep onset latency and shorter total sleep time were associated with a higher likelihood of suicidal thoughts the following day. These findings suggest that disturbances in sleep patterns may function as proximal risk factors for suicidal ideation and represent potential targets for early intervention in suicidal youth (57).

Compared with traditional clinical assessments, which are often retrospective and delayed, AI-based monitoring may provide continuous, real-time, and more objective data on mental health indicators. International studies suggest that such approaches may help identify short-term risk and complement conventional clinical evaluations. However, ethical considerations must remain central. While AI cannot replace human contact or clinical judgement and may generate false alarms, it may serve as a complementary tool for detecting short-term risk windows and enabling timely, tailored interventions.

The emerging literature on AI and suicide prevention can broadly be grouped into three areas: passive sensing and wearable technologies, machine learning models for risk prediction, actigraphy, and ecological momentary assessment combined with AI.

## Conceptual framework and conceptual model

Taken together, the literature suggests that suicide risk may be shaped by the interaction of several short-term and contextual factors framed within the concept of symbiotic intelligence. Large-scale shock events and intense media coverage may create heightened psychological stress within the population (61), particularly among vulnerable individuals. In highly digitalised environments, such events may also be amplified through social media, where exposure can be repeated, emotionally intense, and difficult to regulate.

Systematic reviews further indicate that social media environments may influence risk behaviors among adolescents and young adults. This includes exposure to self-harm-related content and alcohol-related norms. Alcohol use has also consistently been associated with increased suicidality, partly through reduced inhibition, increased impulsivity, and intensified emotional distress.

Within this broader context, individuals experiencing psychological strain may be exposed simultaneously to several reinforcing risk factors, including high social media engagement, alcohol use, and stressful societal events. These interacting dynamics may contribute to short-term “risk windows” in which suicidal behavior becomes more likely.

AI-based interdisciplinary approaches may therefore offer a complementary opportunity to detect early signals of escalating distress. Such approaches may analyse behavioral patterns, language use, physiological signals, and self-reported indicators in near real time. These emerging technologies may provide an additional layer of situational awareness in suicide prevention. When integrated responsibly within existing health systems, such tools may help clinicians and public health services recognize early signs of escalating distress and intervene before crises develop.

**Figure 2:**
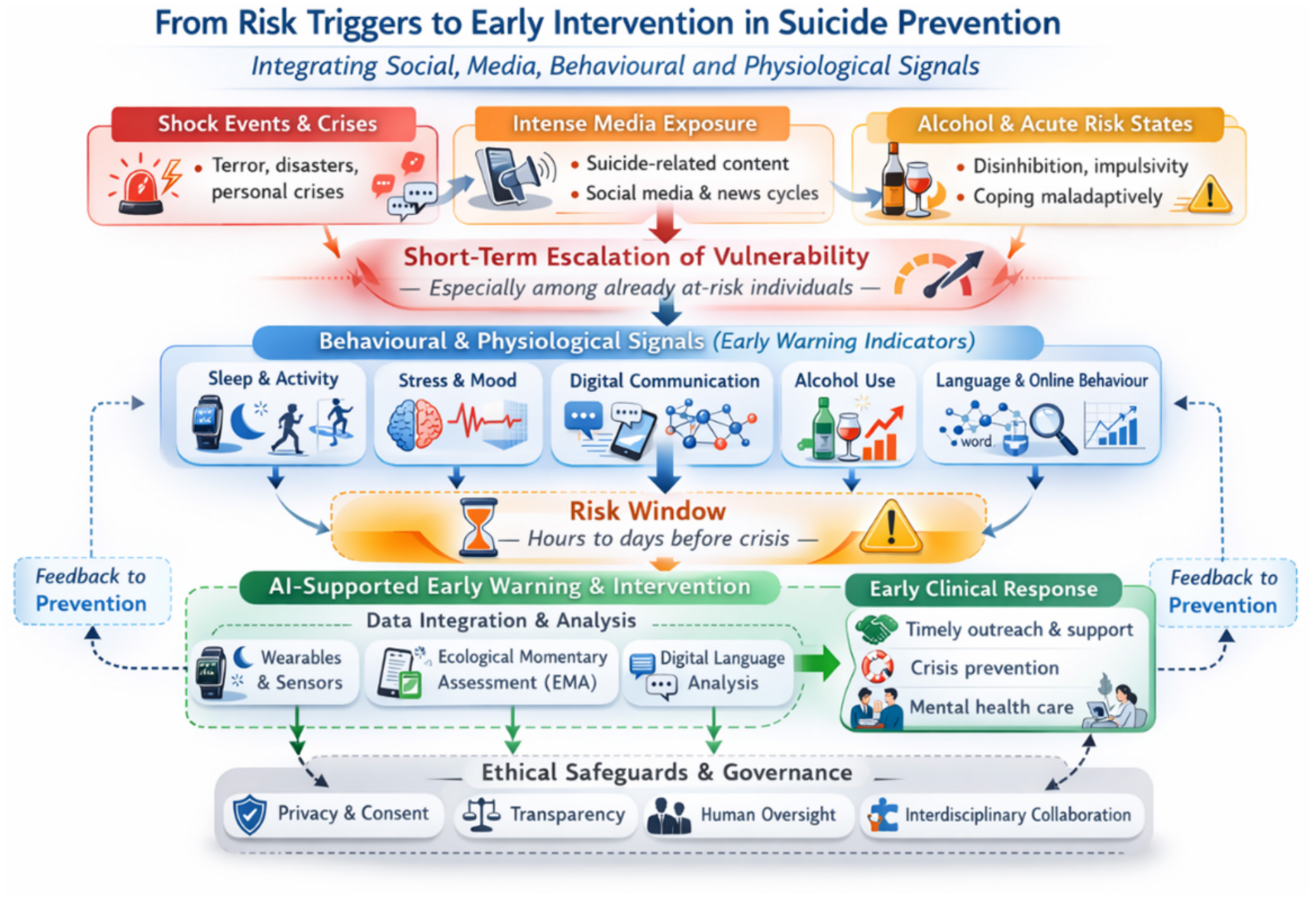
Conceptual model of interacting risk factors and AI-supported early warning. Conceptual model illustrating how societal shock events, media exposure, social media environments, alcohol use, and psychological distress may interact to create short-term suicide risk windows. AI-supported monitoring systems, integrating information about behavioral and physiological signals, may contribute to early detection of risk windows and spur a concomitant early clinical response.

The conceptual model is illustrated in Figure 2. Suicide risk may emerge through the interaction of societal shocks, digital environments, and behavioral risk factors. AI-supported monitoring systems may therefore offer a complementary tool for detecting emerging short-term risk windows and enabling an earlier clinical response.

However, achieving this may require moving beyond more conventional research designs toward AI-supported methods capable of improving measurement precision, validity, and reliability in the analysis of complex digital behaviors (Figure 1). The model is not intended as a predictive framework. Rather, it should be understood as an interdisciplinary heuristic tool for identifying potential points of intervention within complex and rapidly evolving digital environments.

## Clinical implementation

Artificial intelligence is already being implemented in hospitals and clinical settings through integration into clinical decision-making, hospital operations, medical imaging, and real-time patient monitoring via wearable technologies. AI currently contributes to disease detection and clinical support across multiple medical disciplines (58), although important challenges related to data quality, bias, interpretability, and ethical governance remain unresolved. Within mental health care, a recent systematic review by Abdelmoteleb et al. (59) suggests that AI-powered tools integrated into health systems show promising potential for improving the prediction of suicide risk and suicide attempts.

However, practical implementation within suicide prevention differs fundamentally from implementation in many other medical domains. Suicide is a low-base-rate phenomenon, and prediction in psychiatry remains inherently uncertain. Even AI models demonstrating relatively high predictive accuracy may, when deployed at scale, generate a substantial number of false positives. At the system level, this may contribute to clinician alert fatigue, increased workload, unnecessary escalation procedures, and additional pressure on already resource-constrained mental health services. From the patient perspective, repeated false alarms or overly intrusive monitoring may also increase anxiety, stigma, mistrust, or feelings of being under surveillance.

For this reason, AI-supported suicide prevention should not be understood as an autonomous prediction system or a replacement for clinical judgement. Rather, within a symbiotic intelligence framework, AI-derived signals may function as an additional layer of situational awareness that supports human interpretation and clinical reflection. The goal is therefore not automated intervention, but earlier recognition of rapidly escalating distress within existing therapeutic and clinical pathways.

In practical hospital implementation, such systems could potentially be integrated into existing mental health services, outpatient follow-up systems, emergency psychiatry, university student health services, or post-discharge suicide prevention pathways. Participation would necessarily require informed consent and clear governance structures.

Patients already receiving follow-up for suicidal ideation, self-harm, depression, alcohol-related difficulties, or severe psychological distress could voluntarily use wearable devices, ecological momentary assessment (EMA) systems, or smartphone-based monitoring tools capable of collecting behavioral and physiological information in real time.

Within such a framework, AI-supported systems may analyse temporal changes in sleep patterns, stress levels, heart rate variability, activity levels, circadian disruption, digital communication behavior, self-reported mood, alcohol consumption, or abrupt changes in social withdrawal and online activity. Importantly, the emphasis would not be placed on isolated indicators alone, but rather on the interaction between multiple converging signals over time. Figure 2 illustrates such a conceptual framework, where societal shock events, media exposure, social media environments, alcohol use, and psychological distress may interact dynamically to create short-term suicide risk windows.

A symbiotic clinical response could take several forms depending on context and severity. For example, a moderate short-term risk signal might prompt a clinician to initiate a brief digital or telephone check-in, review recent stressors such as sleep disruption, alcohol use, or shock-related media exposure, or increase the frequency of follow-up contacts temporarily. In cases where individuals are already in treatment, AI-derived information could inform clinical discussions, contextual assessment, or closer observation rather than trigger immediate escalation procedures. In this sense, the AI system would function similarly to an early-warning support tool rather than an independent decision-making system.

At the organizational level, implementation would require interdisciplinary collaboration between clinicians, psychologists, psychiatrists, nurses, AI researchers, ethicists, public health authorities, and legal experts. Pilot implementation projects should therefore initially be small-scale, carefully monitored, and embedded within existing health services. Such pilot studies may help evaluate feasibility, clinical usefulness, false-positive rates, patient acceptance, ethical implications, and organizational consequences before broader implementation is considered.

At the same time, ethical safeguards must remain central. Privacy protection, informed consent, transparency, proportionality, secure data handling, and the right to withdraw participation are essential prerequisites. Algorithmic systems must also be continuously evaluated for potential bias, inequitable outcomes, and unintended consequences, particularly when used among vulnerable populations. The purpose should never be large-scale behavioral surveillance, but rather carefully designed support systems intended to strengthen existing preventive efforts.

The most realistic and ethically defensible role for AI in suicide prevention may therefore not be large-scale automated prediction, but modest and carefully integrated support within a broader symbiotic intelligence framework. When combined with contextual understanding, and therapeutic relationships, AI-supported monitoring systems may help clinicians recognize subtle behavioral and physiological changes earlier than conventional assessments alone. Feasible pilot scenarios may include voluntary monitoring systems integrated into outpatient follow-up care, post-discharge suicide prevention programmes, or university student mental health services. Such approaches may potentially contribute to earlier intervention during short-term “risk windows” while still maintaining the central role of human care, trust, and clinical responsibility in suicide prevention.

Figure 2 illustrates how suicide risk may increase through the interaction of several short-term risk factors and how AI-supported systems potentially may help identify critical “risk windows” before a crisis becomes clinically visible. The model proposes that societal shock events, intense media exposure, social media environments, alcohol use, and psychological distress may reinforce one another and contribute to a temporary escalation of vulnerability, particularly among individuals already at risk.

This escalation may be reflected through observable behavioral and physiological changes such as disturbed sleep, increased stress, mood fluctuations, altered digital communication patterns, increased alcohol consumption, and changes in online language or behavior. Such warning signals may emerge hours or days before an acute suicidal crisis.

Within a symbiotic intelligence framework, AI-supported monitoring systems integrating wearable sensors, ecological momentary assessment (EMA), and digital language analysis may help detect these subtle changes in near real time. The purpose is not to replace clinicians or automatically predict suicide, but to provide an additional layer of situational awareness that may support earlier clinical outreach, closer follow-up, and timely preventive interventions. The model also emphasizes that ethical safeguards—including privacy protection, informed consent, transparency, and human oversight—are essential prerequisites for responsible implementation in healthcare settings. It is important to recognize that AI systems may perform unevenly across demographic groups due to biased training data, unequal digital participation, or cultural differences in language use. Such challenges raise important questions regarding equity, fairness, and the potential risk of under- or over-identification within vulnerable populations.

It is also important to recognize that suicide remains a statistically rare event, creating substantial challenges for predictive modeling. Even highly sensitive AI systems may generate large numbers of false positives when applied at the population level. AI-based approaches should therefore be understood as complementary risk-support systems rather than deterministic prediction tools.

## Conclusion and future directions

Suicide prevention remains one of the most complex challenges in public health. The record-high suicide figures in Norway in 2024 highlight the urgency of strengthening existing prevention strategies while also exploring innovative complementary approaches. The evidence discussed in this position paper suggests that several factors may interact in ways that are not always fully captured by traditional clinical assessment frameworks. Shock events, intense media exposure (63, 64), and alcohol-related acute risk states may all contribute to short-term escalation of vulnerability among individuals who are already at risk.

Research on media effects indicates that suicide-related content may produce both harmful and protective dynamics. The so-called *Werther effect* describes how certain forms of suicide-related media exposure can be associated with increased suicidal behavior, while the *Papageno effect* highlights how narratives emphasizing coping, recovery, and help-seeking may have protective influences. In highly digitalised societies, where individuals are continuously exposed to information flows through social media and online platforms, these mechanisms may operate in subtle ways that are difficult to detect through conventional monitoring systems alone.

At the same time, a growing body of research suggests that behavioral and physiological signals often precede acute deterioration in mental health. Changes in sleep patterns, stress levels, activity, digital communication, and alcohol use may emerge in the hours or days before a crisis becomes clinically visible. Emerging AI-based interdisciplinary approaches that combine passive sensing, actigraphy, ecological momentary assessment, and digital language analysis may therefore offer a complementary opportunity to detect such short-term “risk windows”.

However, these AI-technologies must be approached with caution. Suicide is a low-base-rate phenomenon, and prediction in psychiatry remains inherently uncertain. AI systems cannot replace clinical judgement, therapeutic relationships, or community-based prevention efforts. Ethical safeguards must remain central.

The most promising path forward is therefore not large-scale technological deployment, but interdisciplinary carefully designed pilot projects embedded within existing health services. Such pilots could explore whether AI-supported early-warning systems—integrating behavioral signals, digital communication patterns, and self-reported indicators such as alcohol use—can contribute to earlier recognition of rapidly escalating distress.

Ultimately, suicide prevention will always depend on human relationships, timely care, and a society willing to take responsibility for its most vulnerable members. Artificial intelligence should not be viewed as a solution in itself. Rather, if used responsibly, it may function as a modest but potentially valuable additional layer of situational awareness—helping clinicians and health systems recognize emerging crises earlier and intervene before it is too late.

## Data Availability

The original contributions presented in the study are included in the article/Supplementary material, further inquiries can be directed to the corresponding author.
