# Supplementary material for: Position paper on symbiotic intelligence in healthcare: Can AI help us better understand suicidal behavior and prevent suicide?
Source: Front Med (Lausanne). 2026 Jun 22;13:1848732. doi: 10.3389/fmed.2026.1848732 (PMC13333444; doi:10.3389/fmed.2026.1848732)
Supplement: Supplementary file 1 [file Supplementary_file_1.pdf]

Protocol for a PRISMA-ScR-Informed Scoping Review

Title

Artificial intelligence-supported symbiotic intelligence in suicide prevention: A PRISMA-ScR-informed scoping review protocol on digital behavioural signals, passive sensing, ecological momentary assessment, wearable technologies, and short-term suicide risk windows

---

Background

Suicide remains one of the most complex and persistent challenges in global public health. Worldwide, suicide is among the leading causes of death among adolescents and young adults, and despite substantial prevention efforts, the ability to identify individuals at imminent risk remains limited (Sedgwick et al., 2019). In Norway, suicide rates reached their highest level in 25 years in 2024, further underscoring the need for innovative and interdisciplinary approaches capable of complementing traditional clinical assessment frameworks (Norwegian Institute of Public Health [NIPH], 2025).

A growing body of research suggests that suicide risk may fluctuate dynamically over short periods of time and may be influenced by interactions between psychological vulnerability, alcohol use, sleep disturbances, societal shock events, social media exposure, and digital behaviour (Macrynikola et al., 2021; Purba et al., 2023; Cheng et al., 2024). Such fluctuations may create short-term “risk windows” in the hours or days preceding suicidal crises. However, many of these transient changes remain difficult to identify through conventional retrospective clinical approaches.

Recent advances in artificial intelligence (AI), machine learning, ecological momentary assessment (EMA), passive sensing, wearable technologies, actigraphy, and digital language analysis have generated increasing interest in whether behavioural and physiological signals can contribute to earlier recognition of escalating suicide risk (Shen et al., 2025; Melia et al., 2025; Liu et al., 2025). Emerging evidence suggests that variables such as sleep disruption, changes in activity levels, stress markers, social withdrawal, digital communication patterns, and online language use may provide measurable indicators of acute deterioration in mental health (Kivelä et al., 2024; Ratzon et al., 2024; Smagula et al., 2025).

At the same time, the evidence base remains fragmented across several disciplines, including suicidology, psychiatry, public health, digital health, AI research, media studies, behavioural science, and substance use research. Existing studies vary considerably in design, populations, data sources, AI methodologies, outcomes, ethical frameworks, and implementation contexts. Furthermore, the field is characterised by rapid technological development and significant methodological heterogeneity.

Within this context, a scoping review is particularly appropriate because the aim is not primarily to estimate pooled effect sizes, but rather to systematically map the breadth and characteristics of the literature, identify conceptual and methodological gaps, clarify emerging themes, and inform future empirical research and clinical pilot studies (Arksey & O'Malley, 2005; Levac et al., 2010).

This review is informed by the PRISMA Extension for Scoping Reviews (PRISMA-ScR) framework (Tricco et al., 2018) and methodological guidance from the Joanna Briggs Institute (Peters et al., 2020). In addition, the review is theoretically informed by emerging literature on conceptual and interdisciplinary research synthesis within digital health and AI-supported healthcare (Jaakkola, 2020).

Aim

The aim of this scoping review is to systematically map and synthesise the existing literature examining how artificial intelligence-supported approaches may contribute to identifying short-term suicide risk windows through behavioural, physiological, social media-related, alcohol-related, and digital communication signals within a symbiotic intelligence framework.

Review Questions

The review will address the following questions:

1. What types of AI-supported approaches have been used to identify, monitor, or predict suicidal ideation, suicide attempts, self-harm, or acute psychological deterioration?
2. Which behavioural, physiological, linguistic, social media-related, alcohol-related, or passive sensing indicators have been examined in relation to suicide risk?
3. How are short-term suicide risk windows conceptualised and operationalised across studies?
4. What types of wearable technologies, ecological momentary assessment systems, actigraphy approaches, passive sensing methods, and machine learning models are currently being used?
5. What evidence exists regarding clinical implementation, early-warning systems, and integration into healthcare pathways?
6. What ethical, legal, governance-related, and methodological challenges are identified in the literature?
7. To what extent does the literature support a symbiotic intelligence framework in which AI functions as a complementary support system rather than an autonomous predictive system?

Methodology

Study Design

This study will employ a scoping review methodology informed by the frameworks proposed by Arksey and O'Malley (2005), Levac et al. (2010), and the Joanna Briggs Institute (Peters et al., 2020). Reporting will follow the PRISMA-ScR checklist and explanation document (Tricco et al., 2018).

A scoping review design is considered appropriate because the field is broad, interdisciplinary, rapidly evolving, and characterised by heterogeneous methodologies and conceptual approaches.

Eligibility Criteria

The review will apply the Population–Concept–Context (PCC) framework recommended for scoping reviews (Peters et al., 2020).

Population

The review will include studies involving:

- adolescents,
- young adults,
- university students,
- adults,
- psychiatric populations,
- individuals with suicidal ideation or suicide attempts,
- individuals with self-harm behaviour,
- high-risk populations,
- and general population samples where suicide-related outcomes are examined.

Concept

The review will include studies examining:

- artificial intelligence,
- machine learning,
- deep learning,
- natural language processing,
- digital phenotyping,
- passive sensing,
- wearable technologies,
- actigraphy,
- ecological momentary assessment (EMA),

- smartphone-based monitoring,
- digital language analysis,
- social media analytics,
- alcohol-related digital indicators,
- AI-supported early-warning systems,
- and symbiotic intelligence approaches related to suicide prevention.

Context

Relevant contexts include:

- mental health services,
- psychiatry,
- emergency care,
- suicide prevention,
- public health monitoring,
- digital health,
- university health services,
- social media environments,
- and technology-supported clinical monitoring.

Inclusion Criteria

Studies will be included if they:

- examine suicidal ideation, suicide attempts, self-harm, suicidal behaviour, or acute suicide risk;
- involve AI-supported or digitally supported monitoring, prediction, or detection methods;
- include wearable, physiological, behavioural, social media, alcohol-related, linguistic, EMA, or passive sensing data;
- are empirical studies, systematic reviews, meta-analyses, scoping reviews, umbrella reviews, protocols, or theoretically relevant conceptual papers;
- are published in peer-reviewed journals;
- are published between January 2010 and December 2025;
- are written in English or Scandinavian languages.

Exclusion Criteria

Studies will be excluded if they:

- focus exclusively on general mental health without suicide-related outcomes;
- discuss AI or digital health without relevance to suicide prevention;

- are editorials, opinion pieces, or commentaries without substantive analytical or theoretical contribution;
- lack methodological description;
- focus exclusively on unrelated neurological or somatic conditions.

Information Sources

The following databases will be searched:

- PubMed/MEDLINE
- PsycINFO
- Scopus
- Web of Science
- CINAHL
- IEEE Xplore
- ACM Digital Library

Additional searches will be conducted through:

- backward citation searching,
- forward citation tracking,
- Google Scholar,
- and relevant grey literature sources from WHO, public health institutes, and suicide prevention organisations.

Search Strategy

The search strategy will combine controlled vocabulary and free-text terms related to suicide prevention, artificial intelligence, passive sensing, social media, wearable technologies, alcohol use, and digital behavioural monitoring.

A preliminary PubMed search string is presented below:

("suicide" OR "suicidal ideation" OR "suicide attempt" OR "self-harm" OR "suicidal behavior")
AND
("artificial intelligence" OR "machine learning" OR "deep learning" OR "natural language processing" OR "digital phenotyping" OR "passive sensing" OR "wearable*" OR "actigraphy" OR "ecological momentary assessment" OR "EMA" OR "social media" OR "digital behavior" OR "digital behaviour")
AND
("prediction" OR "early warning" OR "risk detection" OR "monitoring" OR "risk window" OR "real-time")

Additional thematic searches will target:

- alcohol-related suicide risk,
- media exposure and the Werther/Papageno effects,
- sleep disturbances and suicide risk,
- passive sensing and wearable technologies,
- EMA and machine learning approaches.

The search strategy will be iteratively refined in consultation with a research librarian.

Study Selection

All identified references will be imported into EndNote and subsequently uploaded into Covidence for screening and duplicate removal.

Two independent reviewers will conduct:

1. title and abstract screening,
2. full-text screening.

Disagreements will be resolved through discussion or consultation with a third reviewer.

The study selection process will be documented using a PRISMA flow diagram (Tricco et al., 2018).

Data Extraction

A standardised data charting form will be developed and piloted.

The following information will be extracted:

- author(s),
- publication year,
- country,
- study design,
- sample characteristics,
- suicide-related outcomes,
- AI or machine learning methods,
- wearable or sensing technologies,
- social media or digital indicators,
- alcohol-related indicators,
- EMA variables,
- predictive outcomes,
- model performance,

- ethical considerations,
- implementation context,
- limitations,
- and relevance to symbiotic intelligence frameworks.

Data Synthesis

Data will be synthesised narratively and thematically.

The literature will initially be mapped into the following domains:

1. Machine learning models for suicide prediction
2. Passive sensing and wearable technologies
3. Ecological momentary assessment and digital phenotyping
4. Social media exposure and digital communication analysis
5. Alcohol-related suicide risk monitoring
6. Sleep, actigraphy, and physiological markers
7. Clinical implementation and AI-supported early warning
8. Ethical, legal, and governance considerations

The review will also develop a conceptual evidence map illustrating how different digital and behavioural indicators may contribute to short-term suicide risk detection within a symbiotic intelligence framework.

Quality Appraisal

Consistent with scoping review methodology, formal risk-of-bias assessment will not be used as an exclusion criterion (Peters et al., 2020). However, methodological limitations will be charted descriptively, including:

- sample size,
- external validation,
- generalisability,
- interpretability,
- algorithmic transparency,
- data governance,
- and ethical safeguards.

Ethics and Dissemination

As the review uses publicly available published literature, ethical approval is not required.

Findings will be disseminated through:

- peer-reviewed publication,
- conference presentations,
- and future interdisciplinary pilot projects examining AI-supported early-warning systems embedded within mental health services.

Expected Contribution

This scoping review will provide one of the first comprehensive interdisciplinary mapping of AI-supported, behavioural, physiological, and digital approaches to identifying short-term suicide risk windows within a symbiotic intelligence framework. The review is expected to clarify the current state of the evidence, identify major methodological and ethical gaps, and inform future clinical pilot studies and implementation research within digital mental health and suicide prevention.

References

Arksey, H., & O'Malley, L. (2005). Scoping studies: Towards a methodological framework. *International Journal of Social Research Methodology, 8*(1), 19–32. https://doi.org/10.1080/1364557032000119616

Cheng, B., Lim, C. C. W., Rutherford, B. N., Huang, S., Ashley, D. P., Johnson, B., et al. (2024). A systematic review and meta-analysis of the relationship between youth drinking, self-posting of alcohol use and other social media engagement (2012–21). *Addiction, 119*(1), 28–46. https://doi.org/10.1111/add.16304

Jaakkola, E. (2020). Designing conceptual articles: Four approaches. *AMS Review, 10*, 18–26. https://doi.org/10.1007/s13162-020-00161-0

Kılıç, N., Taşcı, G., Kaya, Ş., & Özsoy, F. (2024). Evaluation of peripheral inflammatory parameters of cases with suicide attempts. *Journal of Psychiatric Research, 175*, 368–373. https://doi.org/10.1016/j.jpsychires.2024.04.018

Levac, D., Colquhoun, H., & O'Brien, K. K. (2010). Scoping studies: Advancing the methodology. *Implementation Science, 5*, Article 69. https://doi.org/10.1186/1748-5908-5-69

Liu, L., Li, Z., Hu, Y., Li, C., He, S., Zhang, S., Gao, J., Zhu, H., & Huang, G. (2025). Predictive performance of machine learning for suicide in adolescents: Systematic review and meta-analysis. *Journal of Medical Internet Research, 27*, e73052. https://doi.org/10.2196/73052

Macrynikola, N., Auad, E., & Miranda, R. (2021). Does social media use confer suicide risk? A systematic review of the literature. *Computers in Human Behavior Reports, 3*, 100094. https://doi.org/10.1016/j.chbr.2021.100094

Melia, R., Musacchio Schafer, K., Rogers, M. L., Wilson-Lemoine, E., & Joiner, T. E. (2025). The application of AI to ecological momentary assessment data in suicide research: Systematic review. *Journal of Medical Internet Research, 27*, e63192. https://doi.org/10.2196/63192

Norwegian Institute of Public Health. (2025). *Dette døde vi av i 2024* [What we died from in 2024]. https://www.fhi.no/nyheter/2025/dette-dode-vi-av-i-2024

Peters, M. D. J., Marnie, C., Tricco, A. C., Pollock, D., Munn, Z., Alexander, L., McInerney, P., Godfrey, C. M., & Khalil, H. (2020). Updated methodological guidance for the conduct of scoping reviews. *JBI Evidence Synthesis, 18*(10), 2119–2126. https://doi.org/10.11124/JBIES-20-00167

Purba, A. K., Thomson, R. M., Henery, P. M., Pearce, A., Henderson, M., Katikireddi, S. V., et al. (2023). Social media use and health risk behaviours in young people: Systematic review and meta-analysis. *BMJ, 383*, e073552. https://doi.org/10.1136/bmj-2022-073552

Ratzon, R., Reiter, J., Goltser-Dubner, T., et al. (2024). Sleep measures as a predictor of suicidal ideation among high-risk adolescents. *European Child & Adolescent Psychiatry, 33*, 2781–2790. https://doi.org/10.1007/s00787-023-02358-7

Sedgwick, R., Epstein, S., Dutta, R., & Ougrin, D. (2019). Social media, internet use and suicide attempts in adolescents. *Current Opinion in Psychiatry, 32*(6), 534–541. https://doi.org/10.1097/YCO.0000000000000547

Shen, S., Qi, W., Zeng, J., Li, S., Liu, X., Zhu, X., et al. (2025). Passive sensing for mental health monitoring using machine learning with wearables and smartphones: A scoping review. *Journal of Medical Internet Research, 27*, e77066. https://doi.org/10.2196/77066

Smagula, S. F., Zhang, G., Albert, S., Lim, S., Harvey, A. G., Irwin, M. R., et al. (2025). Actigraphy-measured sleep/wake characteristics associated with suicidal ideation in older adults who have depression and high suicide risk. *Journal of Clinical Psychiatry, 86*(3), 24m15522. https://doi.org/10.4088/JCP.24m15522

Tricco, A. C., Lillie, E., Zarin, W., O'Brien, K. K., Colquhoun, H., Levac, D., et al. (2018). PRISMA extension for scoping reviews (PRISMA-ScR): Checklist and explanation. *Annals of Internal Medicine, 169*(7), 467–473. https://doi.org/10.7326/M18-0850
